# Supplementary material for: Analysis of HPV-Positive and HPV-Negative Head and Neck Squamous Cell Carcinomas and Paired Normal Mucosae Reveals Cyclin D1 Deregulation and Compensatory Effect of Cyclin D2
Source: Cancers (Basel). 2020 Mar 26;12(4):792. doi: 10.3390/cancers12040792 (PMC7226528; doi:10.3390/cancers12040792)
Supplement: Supplementary file 1 [file cancers-12-00792-s001.zip › cancers-716167-final-supp/supplementary_captions_proofs.docx]

Supplementary file captions

**Supplementary Figure S1.** Overall survival (OS) and disease-free survival analysis (DFS) of groups stratified according to HPV status, tumor site and *CCND1* and *CCND2* expression. **(a)** Kaplan-Meier plots of DFS for HPV(-) and HPV(+) groups (n = 82), **(b)** Kaplan-Meier plot of OS for HPV(-) and HPV(+) groups of patients with the base of the tongue tumors (n = 15, there was no follow-up for one patient, see Table 1 in the main text); (c) Kaplan-Meier plots of OS stratified according to *CCND1* gene deregulation in HPV(-) group; **(c)** Kaplan-Meier plots of DFS stratified according to *CCND1* gene deregulation in HPV(-) group; **(d)** Kaplan-Meier plots of DFS stratified according to *CCND1* gene deregulation in HPV(+) group; **(e)** Kaplan-Meier plots of DFS stratified according to *CCND2* gene deregulation in HPV(-) group; **(f)** Kaplan-Meier plots of DFS stratified according to *CCND1* gene deregulation in HPV(+) group. Tick marks and crosses indicate right censoring.

**Supplementary Table S1.** Summary of clinical data for the present cohort. For four patients, only samples from normal mucosa were available, and thus the HPV status was not determined. Statistical significance gauges association between a variable and the HPV subtypes: (n.s.) non-significant, (*) p < 0.05, (***) p < 0.001. Percentages are column-wise by default, row-wise if “r” is behind the value. NA – not available.

**Supplementary Table S2.** Survival analyses in the present dataset. Patients were clustered according to deregulation of D-type cyclin expression in paired samples. Thresholds for deregulation were following: not (0.5 < FC < 2), up (FC > 2), down (FC < 0.5). Survival events are "death" for OS, and "exitus of the disease" or "recidive" for DFS. HR – hazard ratio, NA – not available.

**Supplementary Table S3.** Survival analyses in the TCGA dataset. Only HPV(-) patients. Patients were clustered according to deregulation of D-type cyclin expression in paired samples. Thresholds for deregulation were following: not (0.5 < FC < 2), up (FC > 2), down (FC < 0.5). Survival events are "death" for OS, and "recidive" for DFS. HR – hazard ratio, NA – not available.
