# Supplementary material for: Analysis of HPV-Positive and HPV-Negative Head and Neck Squamous Cell Carcinomas and Paired Normal Mucosae Reveals Cyclin D1 Deregulation and Compensatory Effect of Cyclin D2
Source: Cancers (Basel). 2020 Mar 26;12(4):792. doi: 10.3390/cancers12040792 (PMC7226528; doi:10.3390/cancers12040792)
Supplement: Supplementary file 1 [file cancers-12-00792-s001.zip › cancers-716167-final-supp/Suppl_Fig1.pdf]

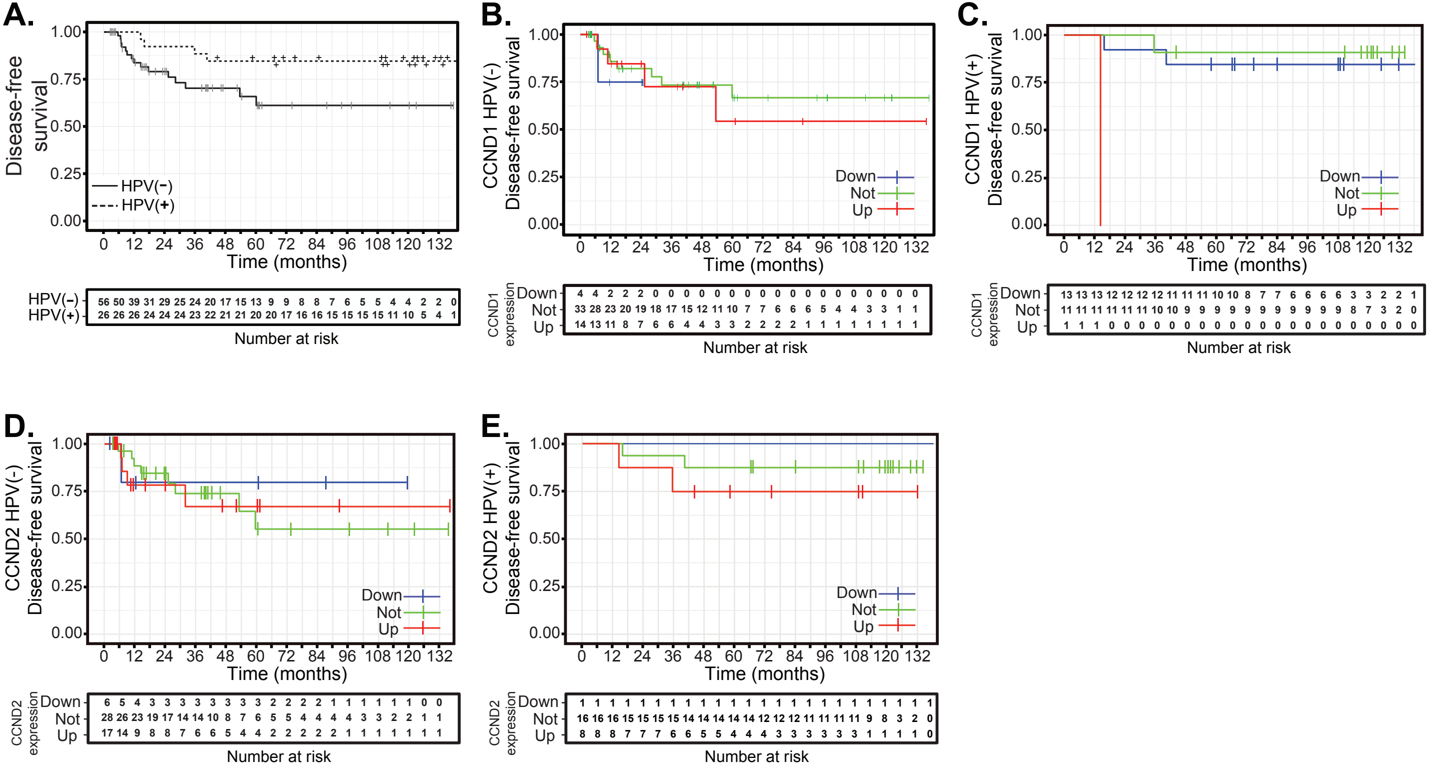
A.
Disease-free survival
1.00
0.75
0.50
0.25
0.00
HPV(–)
HPV(+)
0 12 24 36 48 60 72 84 96 108 120 132
Time (months)
HPV(–) 56 50 39 31 29 25 24 20 17 15 13 9 9 8 8 7 6 5 5 4 4 2 2 0
HPV(+) 26 26 26 24 24 24 23 22 21 21 20 20 17 16 16 15 15 15 15 11 10 5 4 1
Number at risk
B.
CCND1 HPV(-) Disease-free survival
Down
Not
Up
Time (months)
CCND1 expression
Down 4 4 2 2 2 0 0 0 0 0 0 0 0 0 0 0 0 0 0 0 0 0 0 0
Not 33 28 23 20 19 18 17 15 12 11 10 7 7 6 6 6 5 4 4 3 3 3 1 1
Up 14 13 11 8 7 6 6 4 4 3 3 2 2 2 2 2 1 1 1 1 1 1 1 1
Number at risk
C.
CCND1 HPV(+) Disease-free survival
Down
Not
Up
Time (months)
CCND1 expression
Down 13 13 13 12 12 12 12 11 11 11 10 10 8 7 7 6 6 6 6 3 3 2 2 1
Not 11 11 11 11 11 11 10 10 9 9 9 9 9 9 9 9 9 9 9 8 7 3 2 0
Up 1 1 1 0 0 0 0 0 0 0 0 0 0 0 0 0 0 0 0 0 0 0 0 0
Number at risk
D.
CCND2 HPV(-) Disease-free survival
Down
Not
Up
Time (months)
CCND2 expression
Down 6 5 4 3 3 3 3 3 3 3 2 2 2 2 1 1 1 1 1 1 0 0
Not 28 26 23 19 17 14 14 10 8 7 6 5 5 4 4 4 4 3 3 2 2 1 1
Up 17 14 9 8 8 7 6 6 5 4 4 2 2 2 2 2 1 1 1 1 1 1 1
Number at risk
E.
CCND2 HPV(+) Disease-free survival
Down
Not
Up
Time (months)
CCND2 expression
Down 1 1 1 1 1 1 1 1 1 1 1 1 1 1 1 1 1 1 1 1 1 1 1 1
Not 16 16 16 15 15 15 15 14 14 14 14 14 12 12 12 11 11 11 11 9 8 3 2 0
Up 8 8 8 7 7 7 6 6 5 5 4 4 4 3 3 3 3 3 3 1 1 1 1 0
Number at risk
